# Economic evaluation of Motor Neuron Diseases: a nationwide cross-sectional analysis in Germany

**DOI:** 10.1007/s00415-023-11811-1

**Published:** 2023-06-25

**Authors:** Felix Heinrich, Isabell Cordts, René Günther, Benjamin Stolte, Daniel Zeller, Carsten Schröter, Ute Weyen, Martin Regensburger, Joachim Wolf, Ilka Schneider, Andreas Hermann, Moritz Metelmann, Zacharias Kohl, Ralf A. Linker, Jan Christoph Koch, Florentine Radelfahr, Erik Schönfelder, Pavel Gardt, Tara Mohajer-Peseschkian, Alma Osmanovic, Thomas Klopstock, Johannes Dorst, Albert C. Ludolph, Oliver Schöffski, Matthias Boentert, Tim Hagenacker, Marcus Deschauer, Paul Lingor, Susanne Petri, Olivia Schreiber-Katz

**Affiliations:** 1 Department of Neurology, Hannover Medical School, Carl-Neuberg Straße 1, 30625 Hannover, Germany; 2 Department of Neurology, Klinikum Rechts Der Isar, Technical University of Munich, 81675 Munich, Germany; 3 Department of Neurology, University Hospital Carl Gustav Carus, Technische Universität Dresden, 01307 Dresden, Germany; 4 German Center for Neurodegenerative Diseases (DZNE), 01307 Dresden, Germany; 5 Department of Neurology, University Medicine Essen, 45147 Essen, Germany; 6 Department of Neurology, University of Würzburg, 97080 Würzburg, Germany; 7 Hoher Meißner Clinic, Neurology, 37242 Bad Sooden-Allendorf, Germany; 8 Department of Neurology, Ruhr-University Bochum, BG-Kliniken Bergmannsheil, 44789 Bochum, Germany; 9 Department of Molecular Neurology, Friedrich-Alexander-University Erlangen-Nürnberg, 91054 Erlangen, Germany; 10 Center for Rare Diseases Erlangen (ZSEER), University Hospital Erlangen, 91054 Erlangen, Germany; 11 Department of Neurology, Diakonissen Hospital Mannheim, 68163 Mannheim, Germany; 12 Department of Neurology, Martin-Luther University Halle/Saale, 06120 Halle, Germany; 13 Department of Neurology, Klinikum Sankt Georg, 04129 Leipzig, Germany; 14 Translational Neurodegeneration Section “Albrecht-Kossel”, Department of Neurology, University Medical Center Rostock, University of Rostock, 18147 Rostock, Germany; 15 German Center for Neurodegenerative Diseases Rostock/Greifswald, 18147 Rostock, Germany; 16 Department of Neurology, University Hospital Leipzig, 04103 Leipzig, Germany; 17 Department of Neurology, University of Regensburg, 93053 Regensburg, Germany; 18 Department of Neurology, University Medicine Göttingen, 37075 Göttingen, Germany; 19 Friedrich-Baur-Institute, Department of Neurology, University Hospital, Ludwig Maximilian University of Munich, 80336 Munich, Germany; 20 Essener Zentrum Für Seltene Erkrankungen (EZSE), Universitätsmedizin Essen, University Hospital Essen, Essen, Germany; 21 Munich Cluster for Systems Neurology (SyNergy), 80336 Munich, Germany; 22 German Center for Neurodegenerative Diseases (DZNE), 80336 Munich, Germany; 23 Department of Neurology, University of Ulm, 89081 Ulm, Germany; 24 German Center for Neurodegenerative Diseases (DZNE), 89081 Ulm, Germany; 25 Chair of Health Management, School of Business, Economics and Society, Friedrich-Alexander University (FAU) Erlangen-Nürnberg, 90403 Nuremberg, Germany; 26 Department of Neurology with the Institute of Translational Neurology, University Hospital Münster, 48149 Münster, Germany; 27 Department of Medicine, UKM Marienhospital, 48565 Steinfurt, Germany

**Keywords:** Motor Neuron Disease (MND), Cost of illness (COI), Health-related Quality of Life (HRQoL), Quality-adjusted life years (QALYs), Socio-economic burden, Cost-utilities

## Abstract

**Background and objectives:**

Motor Neuron Diseases (MND) are rare diseases but have a high impact on affected individuals and society. This study aims to perform an economic evaluation of MND in Germany.

**Methods:**

Primary patient-reported data were collected including individual impairment, the use of medical and non-medical resources, and self-rated Health-Related Quality of Life (HRQoL). Annual socio-economic costs per year as well as Quality-Adjusted Life Years (QALYs) were calculated.

**Results:**

404 patients with a diagnosis of Amyotrophic Lateral Sclerosis (ALS), Spinal Muscular Atrophy (SMA) or Hereditary Spastic Paraplegia (HSP) were enrolled. Total annual costs per patient were estimated at 83,060€ in ALS, 206,856€ in SMA and 27,074€ in HSP. The main cost drivers were informal care (all MND) and disease-modifying treatments (SMA). Self-reported HRQoL was best in patients with HSP (mean EuroQoL Five Dimension Five Level (EQ-5D-5L) index value 0.67) and lowest in SMA patients (mean EQ-5D-5L index value 0.39). QALYs for patients with ALS were estimated to be 1.89 QALYs, 23.08 for patients with HSP and 14.97 for patients with SMA, respectively. Cost-utilities were estimated as follows: 138,960€/QALY for ALS, 525,033€/QALY for SMA, and 49,573€/QALY for HSP. The main predictors of the high cost of illness and low HRQoL were disease progression and loss of individual autonomy.

**Conclusion:**

As loss of individual autonomy was the main cost predictor, therapeutic and supportive measures to maintain this autonomy may contribute to reducing high personal burden and also long-term costs, e.g., care dependency and absenteeism from work.

## Background

Motor Neuron Diseases (MND) are progressive diseases, that include the involvement of either upper, lower or both types of motor neurons [1]. MND are rare diseases with an estimated prevalence of 6.65/100.000—7.69/100,000 [2] and an incidence of 1.55/100,000—1.75/100,000 people per year [2] in the western world and cause severe disability. MND such as Amyotrophic Lateral Sclerosis (ALS) and Spinal Muscular Atrophy (SMA) result in a reduced life expectancy and have recently been estimated to cause a high socio-economic burden [3–5]. Other MND, such as Hereditary Spastic Paraplegia (HSP), do not necessarily influence life expectancy [6]. Patients have to deal with physical, more often than mental, progressive disability in their lives [3] and rapid disease progression alongside the loss of individual autonomy. This leads to a progressive need for intense, multi-disciplinary medical treatment and care [7–9]. While scientific advances have recently led to the approval of causative therapies for SMA [10–12], approved therapeutics in ALS show a rather limited effect on slowing down disease progression [9]. New therapeutic agents such as sodium phenylbutyrate/taurusodiol are currently evaluated regarding their effect on disease progression (NCT05021536), while in phase II trials [13, 14] first encouraging results were reported. Nevertheless, treatment of most MND still consists of the best supportive care and the alleviation of symptoms [9]. Innovative therapies in rare diseases are associated with high costs, so that cost of illness (COI) studies are mandatory to serve as a basis for additional cost–benefit evaluation [15].

This study aims to examine the socio-economic burden and Health-Related Quality of Life (HRQoL) of MND in Germany, based on real-world primary patient-reported data. As socio-economic evaluations might be driven by economic interests, this study aims to provide an independent analysis. It will depict the current therapeutic landscape and provide evidence for healthcare professionals regarding the best and most cost-effective treatment. This will include reference to current, state-of-the-art therapies, while considering the highest achievable HRQoL. Furthermore, it provides source data for healthcare decision makers regarding the evaluation of future therapeutic options and can serve as a preliminary analysis of the cost-effectiveness of care in an advanced health system.

## Methods

### Patient recruitment and data collection

Patient recruitment for this exploratory multicenter cross-sectional study took place at 17 centers within the German Network for Motor Neuron Diseases (MND Net) [16], between August 2018 and March 2020. 404 patients were enrolled with the majority of patients suffering from ALS (*n* = 325), followed by SMA (*n* = 37) and HSP (*n* = 20).

**Fig. 1:**
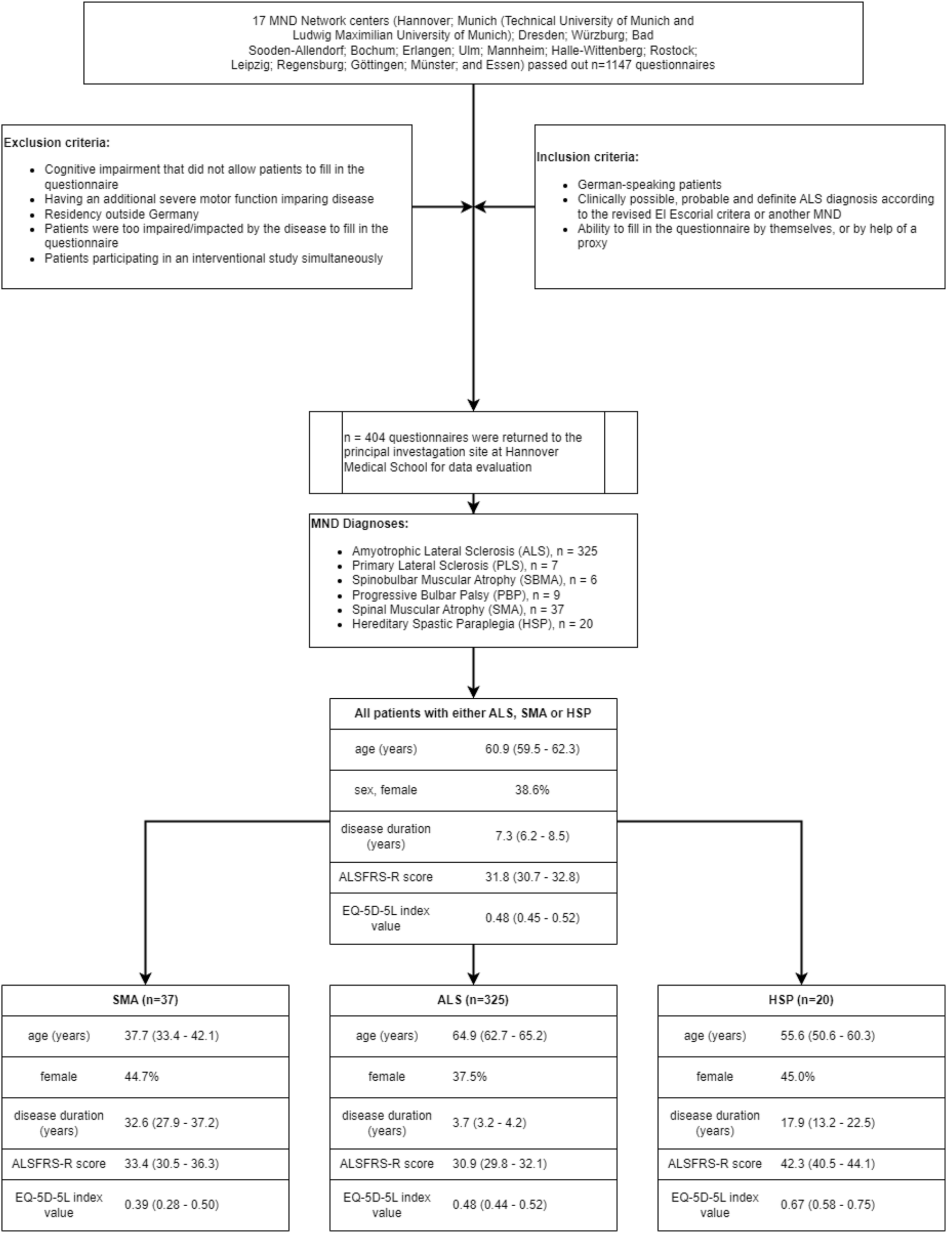
Enrolment criteria and patients’ characteristics. This figure shows the criteria used to select patients for this study and their characteristics. All values are shown as mean values with 95% confidence interval or as percentage and absolute numbers. Diagnosis of ALS was assumed as defined in the El Escorial criteria (Ludolph, A.C.; Drory, V.; Hardiman, O. et al., A revision of the El Escorial criteria 2015. Amyotroph. Lateral Scler. Front. Degener. 2015, 16, 291–292). Abbreviations: ALS = Amyotrophic Lateral Sclerosis, ALSFRS-R = Revised Amyotrophic Lateral Sclerosis Functional Rating Scale (maximum/best = 48 points), EQ-5D-5L index value = EuroQol Group Five Dimension Five Level Scale index value (minimum/worst = -0.205, maximum/best = 1.0), MND = Motor Neuron Disease, n = number

Detailed inclusion and exclusion criteria, patient recruitment rate, and their characteristics are shown in Fig. 1. Using broad inclusion criteria, we aimed to avoid a selection bias.

Patients were enrolled during routine medical visits or they were invited to participate in the study via mail. They were asked to complete a standardized, self-designed, and disease-specific study questionnaire by hand or via proxy assistance. Similar pre-tested questionnaires had been conducted by analyzing disease costs of patients suffering from SMA and Charcot-Marie-Tooth neuropathy [4, 17] and had been used in the analyses of HRQoL and the associated caregiver burden of ALS [18, 19].

The questionnaire assessed primary patient data focusing on individual impairment, self-rated HRQoL, and medical or non-medical resource utilization. To rate patients’ disease severity, the revised Amyotrophic Lateral Sclerosis Functional Rating Scale (ALSFRS-R) with a range of 0 points (total dependence) to 48 points (no impairment in daily activities) [20] was used. Derived from the ALSFRS-R, the patients with ALS were stratified according to the widely used King’s College Staging System [21–23]. Moreover, patients were asked for their care levels, according to the classification within the German nursing care insurance, indicating their need for support in the activities of daily living (care level 1 = mild loss of individual autonomy and consequent need for support, care level 5 = individual autonomy most severely impaired with special demands for nursing care) [24].

Patients’ HRQoL was measured by the EuroQol Group Five Dimension Five Level Scale (EQ-5D-5L) [25]. Depending on responses within the five dimensions (mobility, self-care, usual activities, pain/discomfort, anxiety/depression), the EQ-5D-5L index value was calculated, as recommended by using the German value set (range − 0.205 = worst HRQoL to 1.0 = best HRQoL) [26].

To ensure statistical correctness, all analyses were only performed for MND with over ten participants.

This study report was structured following Strengthening the Reporting of Observational Studies in Epidemiology (STROBE) statement criteria [27].

### Cost estimation

To estimate the socio-economic burden of MND from a societal perspective, we used a micro-costing method according to the latest health-economic recommendations for Germany [28, 29]. Costs were subdivided into Direct Medical Costs (DMC), Direct Non-Medical Costs (DNMC), and Indirect Costs (IC). Direct costs are paid by the patient, insurance, or by third parties. As our population was set in Germany, the insurance could be either a statutory or a private health care provider.

DMC occur within medical treatments such as doctor consultations, inpatient hospital care, drugs, or supportive devices (e.g., cough-assisted interventions, communication devices, non-invasive ventilation) and are mainly covered by health insurances in Germany. Under special circumstances of a so-called “Individueller Heilversuch” [individualized therapeutic approach] even drugs that are not approved by the European Medical Agency (EMA) but in countries outside the European Union (EU), such as edaravone, may be paid by the (statutory) health insurance in Germany as an off-label treatment. DMC were estimated by valuing the utilization of resources reported by patients. All resources were subdivided into price units that were calculated following established guidelines [28]. For occasions when the latest 2019 data were unavailable, inflation-adjusted [30] working group data for 2018 [3] were substituted. DNMC are mainly costs related to informal care provided by relatives or other non-trained caregivers, travel expenses, investments in constructional alterations, and legal fees [28]. To estimate informal care costs, we applied a substitutional approach and asked the patients to report the time of assistance required from a non-professional caregiver, in hours per day. This time was substituted by the costs that would have occurred if this assistance had been conducted by a professional caregiver.

In contrast, IC occur due to absence from work, invalidity, or premature death [31].

Whenever patients did not provide wage levels, average wage levels for Germany in 2019 [32] were used. We used the human capital approach to value the loss of productivity due to reduced working ability [31].

As recall periods were different for some items, we extrapolated all costs to one year, anticipating a stable resource utilization for every patient over this time, in order to avoid recall bias. All costs were calculated in Euros (€) for 2019 (main enquiry period). Whenever international literature with prices/costs in US Dollar ($) was used, we transferred them to Euros (€) by using the mean exchange rate for 2019 (1 € = 1.1195 US $) [33] to ensure comparability.

### Estimation of Quality-Adjusted Life Years (QALYs)

QALYs are calculated by multiplying a patient’s HRQoL, measured with the EQ-5D-5L index value, with the time until a patient is facing a new constraint in quality of life or death. They can therefore be understood as the area under the curve in a plot of HRQoL against time. If the costs of a therapy are divided by the QALYs, this can offer a measure to compare different therapies [34].

As a rough QALY estimation for ALS, the mean HRQoL per King’s stage was calculated and multiplied by the mean duration of each stage [21]. Afterwards, the QALYs per stage were added and divided by the mean overall cost per patient. As not every individual patient reaches King`s stages 4a and 4b one after the other, but maybe only one or the other (or none) of these, we used a weighted average for them by taking into account the number of patients in each stage.

For SMA and HSP, data are even more limited because no comparable staging systems exist. To estimate QALYs in these diseases, that were calculated based on analytical models in previous reports [35], we assumed a steady state of health until death and used previously reported survival data [35]. HSP generally does not constrain life expectancy and, therefore, the mean life expectancy of a healthy age-matched German person (70.6 years) [36] was used.

### Statistical analysis

Statistical analysis was performed using IBM® Statistical Software Package of Social Science (SPSS®, IBM, Armonk, NY, USA) version 27. Demographic data and descriptive statistics were determined using frequency tables and exploratory data analysis. Based on the target to identify influencing factors for both cost and HRQoL (dependent variables), we defined independent variables according to their clinical relevance. Those were entered into a multiple linear regression model using a stepwise forward selection to identify criteria with the largest effect on the dependent variables. Therefore, an *F*-test was used, including variables with an *F*-value ≤ 0.05 and excluding variables with *p* > 0.1. The comparison of patients with equal disease severity was ensured by including the ALSFRS-R score as a covariate, other common covariates such as sex and age did not show a significant influence on either COI nor HRQoL and were therefore neglected in the further analyses.

As our population of patients with SMA and HSP was too small to obtain reliable results with this type of analysis, the same potential influencing factors were tested using a Wilcoxon–Mann–Whitney Test to determine whether an independent variable had an influence on either costs or HRQoL. Subgroups were defined by the existence of a dichotomous feature (yes/no) or the median value of a variable. To examine whether a variable resulted in an increase or decrease of either costs or HRQoL in patients with SMA or HSP, the difference between the mean values of each subgroup was calculated. Due to the small patient numbers, we only reported tendencies (below or above zero) for these analyses. A p-value of ≤ 0.05 (two-tailed) was considered statistically significant. Patients with missing values were excluded from all analyses based on respective scores or questionnaires. Only missing values in the ALSFRS-R were replaced. Whenever one value per dimension was missing, we replaced it with the mean of the other values for that particular dimension. Patients who did not fill in any value in one dimension were excluded from all analyses based on the ALSFRS-R.

## Results

### Patients’ characteristics

Patients’ characteristics are shown in Fig. 1 and Table 1. In total, 404 patients were included in this analysis. Patients with ALS represented the greatest cohort that was representative according to age, sex, subtype, and regional distribution throughout all participating centers [18]. The mean ALSFRS-R score was 30.9 in patients with ALS and highest in patients with HSP (42.3), while the mean disease duration from symptom onset differed from 3.7 (ALS) to 32.6 years (SMA) (Fig. 1). Accounting for the mean age (37.7 years) of our SMA study population, most patients demonstrated a later-onset SMA (disease onset at age > 6 months) [37]. Responses showed that only 32.7% of our total patient population faced minor restraints in their autonomy as they did not have a care level (Table 1). Patients with impaired autonomy were moderately to severely affected (51.3% with care levels 3–5), while 38.1% needed the permanent attendance of a caregiver. Evidence supported that 28.5% of all patients had lost their ability to work due to their disease. Additionally, 18.7% of the patient population faced a loss of income due to reduced working ability. Health-related costs were primarily paid by insurances or third parties, but patients spent 7.9% of the total COI themselves (data not shown).

**Table 1:** Demographical data and use of resources of the healthcare system

Characteristics	Percentage or mean (95% CI)			
	All patients (*n* = 404)	ALS (*n* = 325)	SMA (*n* = 37)	HSP (*n* = 20)
Age at disease onset, years (*n* = 399)	53.4 (51.2—55.5)	60.2 (58.5—61.5)	5.2 (2.4—7.9)	36.0 (30.0—41.9)
Body mass index (*n* = 402)	24.0 (23.6—24.5)	24.1 (23.7—24.6)	22.3 (20.2—24.3)	25.6 (23.9—27.3)
Health insurance, statutory (*n* = 404)	85.6	85.5	92.1	75.0
King’s stage (*n* = 325)				
1		17.5		
2		21.5		
3		25.5		
4a		7.4		
4b		28.0		
Care level (*n* = 382)				
None	32.7	30.6	34.2	65.0
1	2.4	2.2	0.0	10.0
2	13.6	13.9	10.2	15.0
3	19.1	20.7	13.2	5.0
4	17.0	17.6	21.1	0.0
5	15.2	15.1	21.1	5.0
Permanent (24/7) attendance of a caregiver necessary (*n* = 382)	38.1	40.3	37.8	5.0
Time consumption for informal care per month, hours (*n* = 404)	251 (209—292)	260 (223—295)	104 (22—194)	389 (-99—945)
Occupational situation (*n* = 365)				
Currently working	23.6	16.2	59.5	70.0
Unemployable due to disease	28.5	32.1	10.8	5.0
Retired, unemployed, student, et cetera	47.9	51.6	29.7	25.0
Needed to change job	8.5	9.8	5.6	7.7
Loss of income	18.7	20.9	15.8	15.4
Use of… (*n* = 404)				
Drugs	86.4	91.7	47.4	75.0
Outpatient physician consultations	87.2	89.2	71.1	85.0
Inpatient hospital treatment	44.6	44.6	65.8	5.0
Informal care	77.3	80.0	65.8	55.0
Professional outpatient care	39.7	39.4	50.0	25.0
Residential or semi-residential care	2.9	2.7	0.0	10.0
Rehabilitation (in- and outpatient)	20.9	21.5	2.6	45.0
Psychological support	9.3	10.5	0.0	5.0
Use of further therapies (*n* = 404)	92.1	94.1	75.5	90.0
Physiotherapy	79.6	80.3	73.7	80.0
Occupational therapy	48.3	54.8	13.2	10.0
Speech therapy	45.2	52.0	5.3	10.0
Respiratory therapy	8.1	9.5	0	0
Others	15.9	16.3	13.2	15.0
Use of supportive devices (*n* = 404)				
Daily living aids	26.2	27.2	26.3	10.0
Mobility aids	68.1	67.8	71.1	75.0
Respiratory aids	25.8	28.3	28.6	0.0
Care aids	55.9	57.4	63.2	20.0
Communication aids	28.2	30.9	18.4	5.0
Feeding tube	11.0	12.9	0	0

Care levels correspond to a classification within the German nursing care insurance, indicating the individual need for support in the activities of dailys living (care level 1 = mild loss of individual autonomy and consequent need for support, care level 5 = individual autonomy most severely impaired with special demands for nursing care) [24]. All percentages were rounded to one decimal place and therefore might not exactly add up to 100. Due to the small number of patients with either SMA or HSP, we did not adjust for outliers and thus the comparability of the informal care provided by caregivers in patients with HSP is limited. Respiratory aids include non-invasive ventilation (*n* = 82/20.3% of all patients) and invasive ventilation via tracheostomy (*n* = 15/3.7% of all patients)

*ALS*  Amyotrophic Lateral Sclerosis, *CI* Confidence Interval, *HSP* Hereditary Spastic Paraplegia, *King’s stage* King’s College Staging System, *n* number, *SMA* Spinal Muscular Atrophy

### Cost estimation

**Fig. 2:**
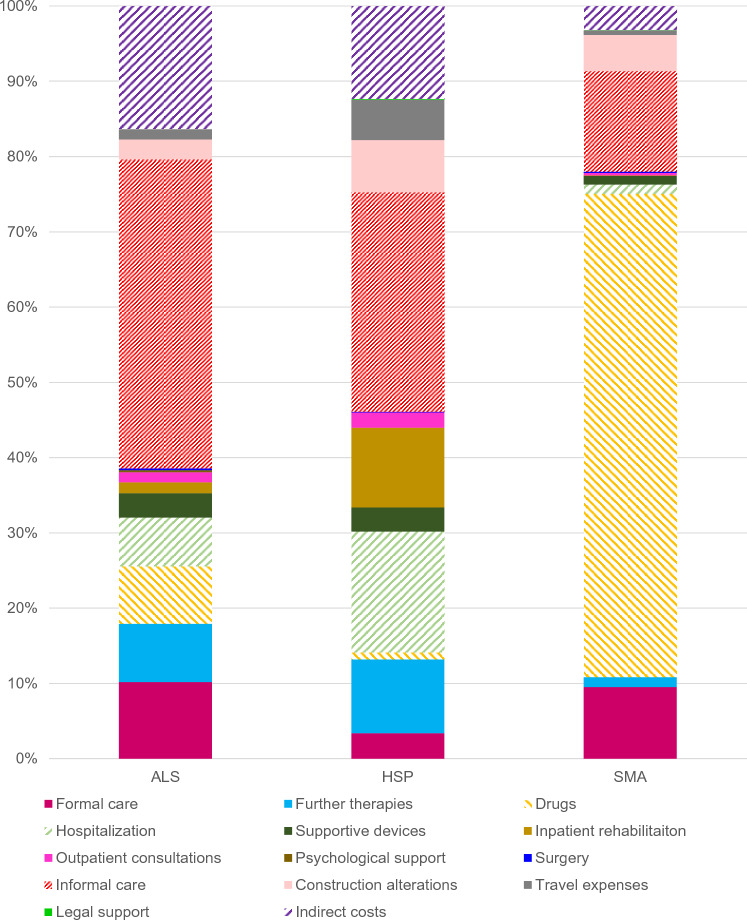
Cost proportions in relation to the total COI. Different MND resulted in different relations of costs as compared to the total COI. Nevertheless, DNMC such as informal care was found to be the major cost driver in ALS and HSP (SMA: drugs). To ensure clarity in this figure we did not number cost proportions under five percent. ALS = Amyotrophic Lateral Sclerosis, COI = Cost of illness, DNMC = Direct Non-Medical Costs, HSP = Hereditary Spastic Paraplegia, MND = Motor Neuron Diseases, SMA = Spinal Muscular Atrophy

**Fig. 3:**
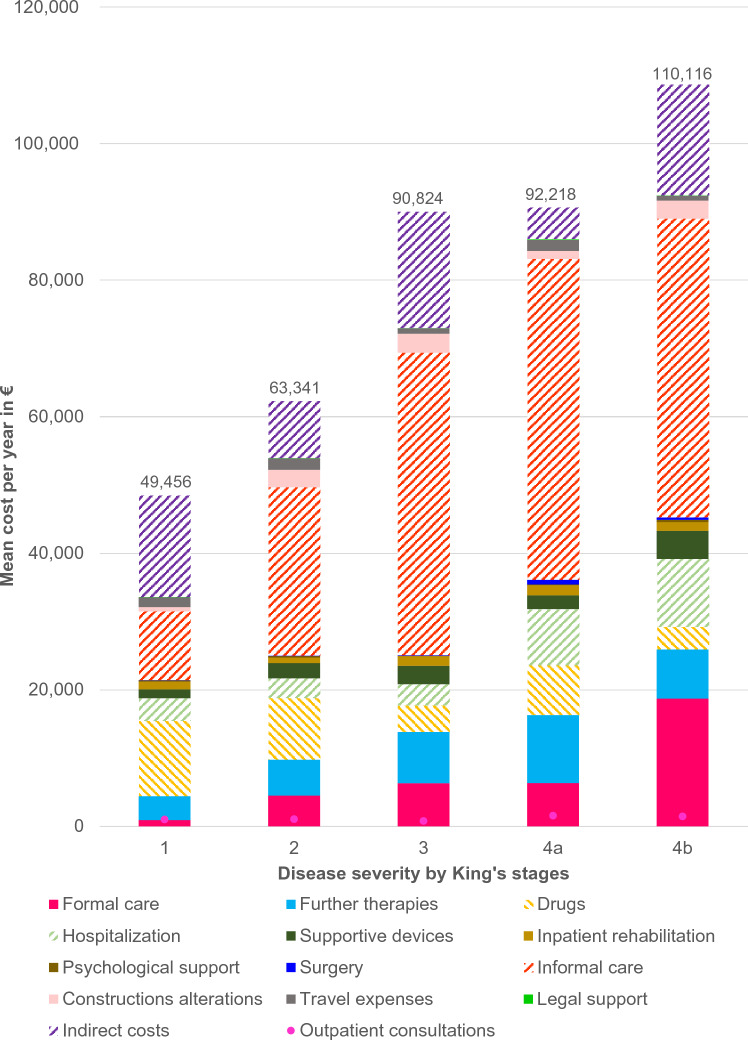
Mean costs per patient with ALS per year in Euros (€) stratified by disease stages**.** We observed increasing costs, especially for formal and informal care, and hospitalization, with an increase of disease severity. ALS = Amyotrophic Lateral Sclerosis, King’s stage = King’s College Staging System

The complete results of our cost analysis are shown in Table 2. Total COI added up to 83,060€ (ALS), 206,856€ (SMA), and 27,074€ (HSP). As shown in Fig. 2, the main cost drivers in patients with ALS and HSP were DNMC and were primarily a result of informal care or disability-adjusted improvements to the living environment (informal care accounting for 41.1% of total COI in patients with ALS and 28.2% of total COI in patients with HSP). The highest costs for SMA were related to drug treatment (66.3% of total COI). This difference can be explained by the high proportion of patients with SMA treated with nusinersen (60.5%, *n* = 23), while only 2.5% (*n* = 8) of the ALS population received more costly drugs such as edaravone. IC contributed to 16.4% (ALS) and 11.9% (HSP) of total COI, respectively. In SMA, IC caused 3.3% of total COI. This shows the different issues that patients are facing in various MND. Results showed that 10.8% of the patients with SMA needed to retire due to their disease progression and 59.5% were currently working, while in ALS only 16.2% of the patients were able to work and 32.1% needed to retire. As shown in Fig. 3, costs related to patients with ALS rose with disease progression. In higher disease stages, informal and formal care costs were responsible for more than 55% of total COI (King’s stage 3 and above). In contrast, costs for drugs were lower with progressive stages of the disease which was in line with the observation that the majority of patients (62.5%) receiving more costly drugs (such as edaravone) were in lower disease stages.

**Table 2:** Total cost of illness (COI) of ALS, SMA and HSP

	ALS			SMA			HSP		
	mean cost per year (€)	(95%-CI, €)	percent of total COI	mean cost per year (€)	(95%-CI, €)	percent of total COI	mean cost per year (€)	(95%-CI, €)	percent of total COI
**DMC**	**33,027**	**(27,140–38,914)**	**39.8**	**166,242**	**(120,213–212,634)**	**80.5**	**12,955**	**(900–25,010)**	**47.9**
Formal care	8450	(4339–12,561)	10.2	20,312	(5051–35,564)	9.8	887	(48–4945)	3.3
Further therapies	6425	(5709–7140)	7.7	2759	(1875–4447)	1.3	2560	(1961–4945)	9.5
Drugs	6327	(3365–9228)	7.6	137,118	(91,479–283,756)	66.3	249	(55–436)	0.9
Hospitalization	5412	(4119–6626)	6.5	2767	(1358–3999)	1.3	4202	(− 4539 to 12,996)	15.5
Supportive devices	2686	(2209–3162)	3.2	2464	(1401–3527)	1.2	840	(143–1537)	3.1
Inpatient rehabilitation	1191	(917–1310)	1.4	154	(− 158–465)	0.1	2776	(1076–4476)	10.3
Outpatient physician consultations	1129	(918–1339)	1.4	711	(388–1033)	0.3	529	(137–920)	2.0
Psychological support	222	(112–333)	0.3	0	0	0.0	0	0	0.0
Surgery	172	(121–224)	0.2	312	(192–432)	0.2	20	(− 11 to 50)	0.1
**DNMC**	**37,488**	**(33,083–41,893)**	**45.1**	**40,217**	**(24,706–55,729)**	**19.4**	**10,886**	**(1638–20,135)**	**40.2**
Informal care	34,122	(29,901–38,341)	41.1	28,527	(13,246–37,719)	13.8	7644	(566–14,723)	28.2
Construction alterations	2176	(1635–2746)	2.6	10,316	(4814–15,817)	5.0	1814	(262–3367)	6.7
Travel expenses	1127	(617–1637)	1.4	1278	(147–2410)	0.6	1395	(− 489–3278)	5.2
Legal support	13	(1–25)	0.0	43	(− 12–98)	0.0	33	(− 36–103)	0.1
**Indirect costs**	**13,583**	**(10,935–16,232)**	**16.4**	**6856**	**(1368–12,360)**	**3.3**	**3233**	**(**− **1896–8362)**	**11.9**
**Total COI**	**83,060**	**(74,919–91,199)**	**100.0**	**206,856**	**(151,357–262,356)**	**100.0**	**27,074**	**(9937–44,617)**	**100.0**

*ALS* Amyotrophic Lateral Sclerosis, *CI* Confidence interval, *COI* Cost of illness, *DMC* Direct Medical Costs, *DNMC* Direct Non-Medical Costs, *HSP* Hereditary Spastic Paraplegia, *SMA* Spinal Muscular Atrophy. As the mean cost per category was calculated from the patient-reported data and not as the aggregation of the mean values of the subcategories (e.g., formal care), percentages do not necessarily add up to 100%

### Health-related quality of life (HRQoL)

Patients with HSP reported the best HRQoL (mean EQ-5D-5L index value 0.67), while patients with SMA registered a lower HRQoL (mean EQ-5D-5L index value 0.39). The HRQoL of patients with ALS was in the middle of these results (mean 0.48).

### Estimation of quality-adjusted life years (QALYs)

Standardized time periods for disease progression in patients with ALS have been previously investigated for predefined clinical stages [21]. Accordingly, patients reach King’s stage 2 after 17.7 months, stage 3 is reached after 23.3 months, stage 4a after 27.7 months, and stage 4b after 30.3 months, from disease onset, while death occurs after an average of 42.3 months. Taking into account the mean EQ-5D-5L index value per King’s stage (data not shown), we were able to calculate a total of 1.89 QALYs for patients with ALS after disease onset. For late-onset SMA, recent research has reported a life expectancy of 38 years from disease onset [35]. Applying the mean EQ-5D-5L index value of 0.39, this added up to 14.82 QALYs left for a patient with SMA at the time of disease onset. HSP is not likely to be associated with premature death [38]; therefore, we calculated the disease duration using the mean general life expectancy in Germany at disease onset, as described above. With a mean disease duration of 34.6 years until death, we estimated a total of 23.18 QALYs for HSP.

Based on the annual COI, cost-utilities were valued at 138,960€/QALY for ALS, 525,044€/QALY for SMA, and 40,573€/QALY for HSP.

### Influencing factors on costs and HRQoL

The results of the linear regression analyses are shown in Tables 3 and 4.

**Table 3:** Linear regression analysis of influencing variables on costs and HRQoL of patients with ALS

	Total costs (COI) (regression coefficient b, 95% CI and std. error in €)				
Independent variable	regression coefficient b	(95% CI)	std. error	T	p-value
Edaravone therapy	171,473	(133,947–209,000)	19,063	8.995	< 0.001
Tracheostomy	60,650	(28,456–92,844)	16,354	3.709	< 0.001
Retirement due to disease	38,851	(25,451–52,250)	6,807	5.708	< 0.001
Permanent attendance of a caregiver necessary	34,347	(19,822–48,472)	7,378	4.655	< 0.001
Use of formal care	25,668	(12,372–38,964)	6,754	3.800	< 0.001
Use of daily living aids	− 16,882	(− 30,207—− 3,558)	6,769	− 2.494	0.013
Disease onset (per year)	− 607	(− 1,109—− 104)	255	− 2.378	0.018
ALSFRS− R score (per increase of one point)	− 2,108	(− 2930—− 1,286)	418	− 5.047	< 0.001

	HRQoL (EQ-5D-5L index value)				
Independent variable	regression coefficient b	(95% CI)	std. error	T	p-value
Use of feeding tube	0.161	(0.086–0.235)	0.038	4.256	< 0.001
Use of speech therapy	0.097	(0.053–0.141)	0.022	4.379	< 0.001
Non-invasive ventilation	0.082	(0.029–0.135)	0.027	3.022	0.003
Retirement due to disease	0.05	(0.003–0.097)	0.024	2.115	0.035
ALSFRS-R score (per increase of one point)	0.016	(0.012–0.020)	0.002	8.342	< 0.001
Care level (per care level)	− 0.032	(− 0.050—− 0.014)	0.009	− 3.442	0.001
Use of informal care	− 0.087	(− 0.174—− 0.028)	0.030	− 2.878	0.004
Permanent attendance of a caregiver necessary	− 0.099	(− 0.154—− 0.044)	0.028	− 3.529	< 0.001
Use of care aids	− 0.174	(− 0.228—− 0.119)	0.028	− 6.302	< 0.001

The following variables were included in the model by their clinical importance: ALSFRS-R score, disease duration, age at disease onset, sex, underweight (body mass index < 20 kg/m^2^), care level, formal care, informal care, permanent attendance of a caregiver necessary, retirement due to disease, need to reduce working hours, self-reported personal impairment due to disease (patients were asked whether they felt impaired in their daily life by the disease), use of daily living aids, use of mobility aids, use of care aids, use of communication aids, non-invasive ventilation, tracheostomy, feeding tube, inpatient hospital care, rehabilitation, number of outpatient physician consultations, use of physiotherapy, occupational therapy, speech therapy, inpatient care, informal care and off-label edaravone therapy. All variables that are not shown in this table had no significant influence on HRQoL and costs. *ALS* Amyotrophic Lateral Sclerosis, *ALSFRS-R* Revised Amyotrophic Lateral Sclerosis Functional Rating Scale, *COI* Cost of illness, *CI* Confidence Interval, *EQ-5D-5L* EuroQoL Five Dimensions Five Levels, *HRQoL* Health-Related Quality of Life,* kg/m*^*2*^ kilograms per square meters, *std.* standard

**Table 4:** Analysis of influencing variables on costs and HRQoL for SMA and HSP patients

	**Total costs (COI)**			
	**SMA**		**HSP**	
Independent variable	p-value	tendency in difference between mean values	p-value	tendency in difference between mean values
Nusinersen therapy	**0.001**	** > 0**	n/a	n/a
Care level	**0.015**	** > 0**	**0.002**	** > 0**
Permanent attendance of a caregiver necessary	**0.026**	** > 0**	**0.033**	n/a
Inpatient hospital care	**0.001**	** > 0**	0.1	n/a
Use of mobility aids	**0.021**	** > 0**	0.081	n/a
Use of care aids	**0.013**	** > 0**	0.249	n/a
Physiotherapy	**0.002**	** > 0**	**0.022**	** > 0**
Use of formal care	0.161	n/a	**0.033**	** > 0**
Use of rehabilitation	0.842	n/a	**0.001**	** > 0**
Less outpatient physician consultations than mean (n = 15/year)	0.935	n/a	**0.002**	** > 0**

	**HRQoL (EQ-5D-5L index value)**			
	**SMA**		**HSP**	
Independent variable	p-value	tendency in difference between mean values	p-value	tendency in difference between mean values
Care level	**0.012**	** < 0**	**0.018**	** < 0**
Permanent attendance of a caregiver necessary	**0.001**	** < 0**	**0.049**	**n/a**
Use of daily living aids	**0.033**	** < 0**	1	n/a
Use of mobility aids	**0.001**	** < 0**	0.163	n/a
Use of care aids	**0.001**	** < 0**	0.426	n/a
Use of communication aids	**0.003**	** < 0**	0.737	n/a
Use of formal care	0.053	n/a	**0.018**	** < 0**
Less outpatient physician consultations than mean (n = 15/year)	0.052	n/a	**0.006**	** < 0**
Physiotherapy	0.570	n/a	**0.018**	** < 0**

The following independent variables were included in our model by their clinical importance: ALSFRS-R score, disease duration, age at disease onset, sex, underweight (body mass index < 20 kg/m^2^), care level, formal care, informal care, permanent attendance of a caregiver necessary, retirement due to disease, need to reduce working hours, self-reported personal impairment due to disease (patients were asked whether they felt impaired in their daily living by the disease), use of daily living aids, use of mobility aids, use of care aids, use of communication aids, non-invasive ventilation, tracheostomy, feeding tube, inpatient hospital care, rehabilitation, number of outpatient physician consultations, use of physiotherapy, occupational therapy, speech therapy, inpatient care, informal care and nusinersen therapy (SMA only). All variables that are not shown in this table had no significant influence on HRQoL and costs. Significant variables are marked bold. The tendency in difference between mean values was marked as not applicable once a variable either showed no significant influence or the number of patients in one group (with variable/without variable) was smaller than in the other group. Abbreviations:* ALSFRS-R *Revised Amyotrophic Lateral Sclerosis Functional Rating Scale, *COI* Cost of illness,* HSP* Hereditary Spastic Paraplegia,* HRQoL*  Health-Related Quality of Life,* n/a* not applicable,* n*  number, *kg/m*^2^  kilograms per square meters,* SMA* Spinal Muscular Atrophy

First, we focused on the ALS patients’ results. The largest negative impact on HRQoL in ALS was observed when the patients needed to use care aids, as they indicated a more severe disease status. This finding was in line with a higher dependency, as signified by a higher care level, the permanent need of presence of a caregiver, and the need of informal care. These circumstances were associated with a cost increase and reduced HRQoL. A reduction in total COI occurred when patients were equipped with daily living aids, indicating higher independency. Lower disease severity, demonstrated by higher ALSFRS-R scores, was associated with higher HRQoL and lower socio-economic costs, while the need for formal care was also affiliated with higher costs. Retirement due to the disease was connected to higher costs and improved HRQoL. For patients with a comparable disease severity, ventilation (especially non-invasive ventilation) improved HRQoL, without being a relevant cost influencer, while tracheostomy increased disease costs. Moreover, the use of a feeding tube and speech therapy significantly increased the individual HRQoL. Other factors, such as the use of mobility aids (e.g., wheelchairs), showed no significant influence on total COI.

The same variables were included to investigate predictors of costs and HRQoL in SMA and HSP (Table 4). Similar to our findings in ALS, care dependency was associated with decreased HRQoL and increased costs. The same effects were observed as soon as patients with SMA became dependent on the permanent attendance of a caregiver. Comparatively, increased HRQoL and decreased costs were connected to care dependency in patients with HSP. For SMA, the use of aids was affiliated with increased costs and decreased HRQoL, whereas this was not observed for patients with HSP, who seemed to benefit from physiotherapy. On the contrary, physiotherapy was associated with elevated costs, but not HRQoL in patients with SMA. Therapy with nusinersen and inpatient hospital care was associated with elevated costs in SMA but showed no significant influence on HRQoL. Additionally, high costs of patients with HSP were associated with formal care, rehabilitation, and a high number of outpatient physician consultations, while these factors also seemed to enlarge HRQoL. Disease severity, as measured by the ALSFRS-R, had no influence on the costs or HRQoL of patients with either SMA or HSP.

## Discussion

Our study provides an analysis of socio-economic costs and HRQoL for different MND. Estimations of QALYs are rare for ALS and HSP, but can provide important evidence for decision makers regarding upcoming pharmaceutical [13, 39] and non-pharmaceutical therapies of MND. Our data can be understood as an assessment basis for further cost-effectiveness studies and payer negotiations.

This study revealed annual costs per patient of 83,060€ for patients with ALS, 206,856€ for patients with SMA and 27,074€ for patients with HSP. Particularly in ALS, costs notably increased with disease progression while HRQoL decreased. With a total population of 83,166,700 inhabitants in Germany (2019) [40] and a prevalence of 8/100,000 [41] for ALS, 1–2/100,000 [42] for SMA and approximately 3/100,000 [43] for HSP, a total COI of these MND in Germany per year add up to 964,247,432€ (ALS: 552,628,049€, SMA: 344,070,184€, HSP: 67,548,892€). These high costs are mainly driven by informal care, new pharmaceutic treatments, as well as indirect costs.

COI has been previously examined for ALS in Germany in a monocentric study [3]. Compared to this analysis, our cost estimation is slightly higher 83,060€ versus 78,256€ [3]). Besides general price increases, this may be due to the larger patient population representing a wider cross-section of the complete population with ALS in Germany and the enrolment of patients under (more costly) off-label edaravone therapy.

In international research on COI of MND different methods, perspectives and health care systems limit the comparability between studies, e.g., as compared to a recent research from the United Kingdom [23].

In comparison to recent British and German analyses in ALS [3, 23], our patients reported a lower HRQoL. This might result from cultural differences in rating quality of life, but also from our multi-center study design, as it included patients from urban areas with good access to healthcare system resources, and rural areas where the permanent availability of highly specialized therapists might be rare. Moreover, study cohorts’ characteristics like the patient proportion in the different disease stages seem to play an important role in the comparability of HRQL results [44].

The use of care aids was associated with lower HRQoL in our study, even when comparing patients with similar ALSFRS-R scores. A possible explanation might be a high individual dependency that is indicated by such aids. This hypothesis is supported by the additional negative influence of having a care level and the need of informal care on HRQoL. Other previously identified predictors of better HRQoL, such as the use of mobility aids, age or tracheostomy, showed no influence on HRQoL of patients with ALS in this study. This may be explained by different sample sizes and the use of different questionnaires to rate HRQoL. Nevertheless, we were able to confirm other predictors of a higher HRQoL such as the use of non-invasive ventilator support, a feeding tube in order to maintain patients’ alimentation, and the use of speech therapy [18, 45]. Astonishingly, retirement due to ALS was found to be associated with higher HRQoL in our study. This may depict that carrying on a profession may be burdensome in ALS.

Regarding SMA, a recent Swedish study reported the COI of patients undergoing nusinersen treatment based on mathematical models [35]. In our study, we used real-world patient data and included only adults which resulted in slightly lower costs per QALY. This difference can be explained by the fact that our study did not exclusively analyze patients treated with nusinersen, different healthcare systems, and price units. Compared to COI estimations prior to the approval of nusinersen [4], COI for SMA in Germany increased by about 420%, and is not likely to decrease immensely due to the market entry of other disease-modifying treatments like Onasemnogene abeparvovec [46]. In HRQoL analyses of patients with SMA, the EQ-5D-5L questionnaire has rarely been used but has shown reduced HRQoL for patients facing SMA [47]. Our findings are in line with previous research using instruments such as the Health Utilities Index Mark 3 (HUI3) [48], confirming a high constraint of HRQoL in patients with SMA.

Regarding HSP, data on socio-economic costs are very limited. Most research concentrates on patients’ burden [49] and not on total COI. As various causative gene mutations have been revealed in the past years [50], new disease-modifying gene therapies are likely to occur, too. Due to their cost, socio-economic research is needed to provide information for decision makers. With regard to the HRQoL, our study confirmed previous research suggesting that HSP can imply a high burden for affected patients along with a further decrease of HRQoL with increased disease severity [51].

When comparing MND, our study showed broad differences in informal care costs between 41.1% (ALS) and 13.8% (SMA; HSP: 18.2%) of total COI. This is in line with previous findings in other neuromuscular disorders such as Duchenne muscular dystrophy [52], Becker muscular dystrophy [52], and Charcot–Marie–Tooth neuropathies [17]. Different diseases result in distinct restraints of daily living and require different needs of care. Changing care dependency results in varying incremental costs depending on the patient’s age. The incremental need of informal care is larger once the patient is an adult, as healthy children require more care than healthy adults. The time spent for informal care in the current analysis varied from 104 h/month (SMA), over 260 h/month (ALS) to 389 h/month (HSP). This underlines the importance of informal care, which is rather common in Germany. It is mainly provided by relatives, especially spouses or children [53]. Therefore, MND imply a high burden not only for patients, but also caregivers [19, 54] and society. Besides being a major cost driver, factors indicating a high need of care and a progressive loss of autonomy (as the need of the permanent attendance of a caregiver) had a negative influence on HRQoL in all investigated MND. The varying impact of distinct influencing factors on costs and HRQoL underlines the differences between the investigated MND and highlights the need for a specific, supportive, and individualized therapy for each patient.

## Limitations and strengths

A limitation of the study is its focus on one single country. As costs vary in different healthcare systems [35, 55], generalization between countries is limited. As IC resulted in a major cost category, some limitations in their evaluation should be taken into account. As not all patients provided wage levels, we needed to replace missing values with the average wage levels for Germany in 2019. Furthermore, costs regarding premature death were not considered in this cross-sectional analysis. Therefore, our estimation of IC may underestimate the actual IC. On the other hand, we used the human capital approach, that is determined from the loss of production potential, to estimate IC and which is known to overestimate costs [31, 56] as a frictional cost approach was not recommended for the use in basic analyses [56] With some factors leading to over- and others leading to underestimation of IC, we are not able to define, whether IC were over- or underestimated.

We did not take into account the psychological, physical, and financial burden of caregivers [19] which may lead to an underestimation of medical and indirect costs. The need to rely on a replacement cost approach to estimate informal care costs limits the accuracy of its estimation. Another limitation is the need to rely on cost valuation recommendations rather than direct data from health insurances and other payers. Direct cost data from health insurances and other payers are unpublished and protected by privacy law acts. This can, again, lead to either under- or overestimation of real costs. Even though we tried to minimize recall bias by using different recall periods, it may have still occurred and led to cost underestimation. The questionnaires for this study were detailed, including over 120 items. This might have led to a selection bias in favor of more motivated, educated, or less severely affected patients. Due to the cross-sectional design of this study, our estimation of QALYs for MND has to be regarded as an approximation because we did not follow individual patients longitudinally. Further bias may result from the impression that our ALS cohort included mainly slow progressors (based on the mean ALSFRS-R score of 30.9/48 and a mean disease duration of 3.7 years). Future research, including longitudinal analyses and comparisons between different international healthcare systems, is necessary to provide reliable information in the light of future therapies.

Nevertheless, this study included a broad patient population due to the multi-center approach, covering an entire country. By its detailed questionnaires, it covered various influencing factors on costs and HRQoL. Our study is providing real-world patient data after the approval of new disease-modifying drugs such as nusinersen. As all MND are rare diseases, the inclusion of a large patient population, especially without incentives such as an anticipated therapeutic benefit, is difficult to achieve. Therefore, our study included a reasonable patient population. Especially for ALS, the described cohort was representative based on the gender distribution and the coverage of different disease stages. The results of our explorative study confirmed and expanded previous research and provided nationwide data for the socio-economic burden of different MND.

## Conclusion

MND account for high socio-economic costs and high constraints in the HRQoL of patients. Disease progression results in a considerable loss of autonomy and can result in higher costs with reduced HRQoL. As patients and their caregivers are highly impacted, costly therapies can contribute to the reduction of secondary costs and improve HRQoL in the long term. Moreover, we observed supportive therapies such as non-invasive ventilation or speech therapy to increase HRQoL, especially in patients with ALS. Patients should be offered such therapies early within the disease course in order to maintain their independence and a higher HRQoL for as long as possible. Most supportive therapies did not appear to be cost drivers in MND and therefore their access should not be restricted. Furthermore, we introduced the concept of QALYs into the evaluation of state-of-the-art therapy of MND in addition to standard cost–benefit analyses for new therapies. They once more confirmed the burden that patients, caregivers, and the entire society are facing when dealing with MND. Further research to stop or delay disease progression is crucial to reduce this encumbrance. Ideally, this occurs before the loss of individual autonomy, but also prior to the creation of secondary costs during individual care, loss of work productivity, and enhanced caregiving requirements. Based on our research, efforts on these key factors are also likely to decrease socio-economic costs.

## Data Availability

Any unpublished data within this article are available from Hannover Medical School and available for qualified investigators in an anonymized format.

## References

[1] Foster LA, Salajegheh MK (2019). Motor neuron disease: pathophysiology, diagnosis, and management. Am J Med.

[2] Park J, Kim JE, Song TJ (2022) The global burden of motor neuron disease: an analysis of the 2019 global burden of disease study. Front Neurol 13:864339. 10.3389/fneur.2022.864339 10.3389/fneur.2022.864339 PMC9068990 35528743

[3] Schönfelder E, Osmanovic A, Müschen LH, Petri S, Schreiber-Katz O (2020). Costs of illness in amyotrophic lateral sclerosis (ALS): a cross-sectional survey in Germany. Orphanet J Rare Dis.

[4] Klug C, Schreiber-Katz O, Thiele S, Schorling E, Zowe J, Reilich P, Walter MC, Nagels KH (2016). Disease burden of spinal muscular atrophy in Germany. Orphanet J Rare Dis.

[5] Larkindale J, Yang W, Hogan PF, Simon CJ, Zhang Y, Jain A, Habeeb-Louks EM, Kennedy A, Cwik VA (2014). Cost of illness for neuromuscular diseases in the United States. Muscle Nerve.

[6] Murala S, Nagarajan E, Bollu PC (2021). Hereditary spastic paraplegia. Neurol Sci.

[7] Mercuri E, Finkel RS, Muntoni F, Wirth B, Montes J, Main M, Mazzone ES, Vitale M, Snyder B, Quijano-Roy S, Bertini E, Davis RH, Meyer OH, Simonds AK, Schroth MK, Graham RJ, Kirschner J, Iannaccone ST, Crawford TO, Woods S, Qian Y, Sejersen T, Muntoni F, Wirth B, Tiziano FD, Kirschner J, Tizzano E, Topaloglu H, Swoboda K, Laing N, Kayoko S, Prior T, Chung WK, Wu S-M, Montes J, Mazzone E, Main M, Coleman C, Gee R, Glanzman A, Kroksmark A-K, Krosschell K, Nelson L, Rose K, Stępień A, Vuillerot C, Vitale M, Snyder B, Quijano-Roy S, Dubousset J, Farrington D, Flynn J, Halanski M, Hasler C, Miladi L, Reilly C, Roye B, Sponseller P, Yazici M, Hurst R, Bertini E, Tarrant S, Barja S, Bertoli S, Crawford T, Foust K, Kyle B, Rodan L, Roper H, Seffrood E, Swoboda K, Szlagatys-Sidorkiewicz A (2018). Diagnosis and management of spinal muscular atrophy: Part 1: Recommendations for diagnosis, rehabilitation, orthopedic and nutritional care. Neuromuscul Disord.

[8] Finkel RS, Mercuri E, Meyer OH, Simonds AK, Schroth MK, Graham RJ, Kirschner J, Iannaccone ST, Crawford TO, Woods S, Muntoni F, Wirth B, Montes J, Main M, Mazzone ES, Vitale M, Snyder B, Quijano-Roy S, Bertini E, Davis RH, Qian Y, Sejersen T (2018). Diagnosis and management of spinal muscular atrophy: Part 2: Pulmonary and acute care; medications, supplements and immunizations; other organ systems; and ethics. Neuromuscul Disord.

[9] Andersen PM, Abrahams S, Borasio GD, de Carvalho M, Chio A, Van Damme P, Hardiman O, Kollewe K, Morrison KE, Petri S, Pradat PF, Silani V, Tomik B, Wasner M, Weber M (2012). EFNS guidelines on the clinical management of amyotrophic lateral sclerosis (MALS)–revised report of an EFNS task force. Eur J Neurol.

[10] Hoy SM (2017). Nusinersen: first global approval. Drugs.

[11] Hoy SM (2019). Onasemnogene abeparvovec: first global approval. Drugs.

[12] Dhillon S (2020). Risdiplam: first approval. Drugs.

[13] Paganoni S, Macklin EA, Hendrix S, Berry JD, Elliott MA, Maiser S, Karam C, Caress JB, Owegi MA, Quick A, Wymer J, Goutman SA, Heitzman D, Heiman-Patterson T, Jackson CE, Quinn C, Rothstein JD, Kasarskis EJ, Katz J, Jenkins L, Ladha S, Miller TM, Scelsa SN, Vu TH, Fournier CN, Glass JD, Johnson KM, Swenson A, Goyal NA, Pattee GL, Andres PL, Babu S, Chase M, Dagostino D, Dickson SP, Ellison N, Hall M, Hendrix K, Kittle G, McGovern M, Ostrow J, Pothier L, Randall R, Shefner JM, Sherman AV, Tustison E, Vigneswaran P, Walker J, Yu H, Chan J, Wittes J, Cohen J, Klee J, Leslie K, Tanzi RE, Gilbert W, Yeramian PD, Schoenfeld D, Cudkowicz ME (2020). Trial of sodium phenylbutyrate-taurursodiol for amyotrophic lateral sclerosis. N Engl J Med.

[14] Paganoni S, Hendrix S, Dickson SP, Knowlton N, Berry JD, Elliott MA, Maiser S, Karam C, Caress JB, Owegi MA, Quick A, Wymer J, Goutman SA, Heitzman D, Heiman-Patterson TD, Jackson C, Quinn C, Rothstein JD, Kasarskis EJ, Katz J, Jenkins L, Ladha SS, Miller TM, Scelsa SN, Vu TH, Fournier C, Johnson KM, Swenson A, Goyal N, Pattee GL, Babu S, Chase M, Dagostino D, Hall M, Kittle G, Eydinov M, Ostrow J, Pothier L, Randall R, Shefner JM, Sherman AV, Tustison E, Vigneswaran P, Yu H, Cohen J, Klee J, Tanzi R, Gilbert W, Yeramian P, Cudkowicz M (2022). Effect of sodium phenylbutyrate/taurursodiol on tracheostomy/ventilation-free survival and hospitalisation in amyotrophic lateral sclerosis: long-term results from the CENTAUR trial. J Neurol Neurosurg Psychiatry.

[15] National Institute for Health and Clinical Excellence (2012) The guidelines manual. London. https://www.nice.org.uk/process/pmg6/resources/the-guidelines-manual-pdf-2007970804933. Accessed 02.10.2022

[16] German Network for Motor Neuron Diseases (MND Net). http://www.mnd-als.de/html/home?set-language-to=en. Accessed 28.01.2021

[17] Schorling E, Thiele S, Gumbert L, Krause S, Klug C, Schreiber-Katz O, Reilich P, Nagels K, Walter MC (2019). Cost of illness in charcot-marie-tooth neuropathy: results from Germany. Neurology.

[18] Peseschkian T, Cordts I, Günther R, Stolte B, Zeller D, Schröter C, Weyen U, Regensburger M, Wolf J, Schneider I, Hermann A, Metelmann M, Kohl Z, Linker RA, Koch JC, Büchner B, Weiland U, Schönfelder E, Heinrich F, Osmanovic A, Klopstock T, Dorst J, Ludolph AC, Boentert M, Hagenacker T, Deschauer M, Lingor P, Petri S, Schreiber-Katz O (2021). A nation-wide, multi-center study on the quality of life of ALS patients in Germany. Brain Sci.

[19] Schischlevskij P, Cordts I, Günther R, Stolte B, Zeller D, Schröter C, Weyen U, Regensburger M, Wolf J, Schneider I, Hermann A, Metelmann M, Kohl Z, Linker RA, Koch JC, Stendel C, Müschen LH, Osmanovic A, Binz C, Klopstock T, Dorst J, Ludolph AC, Boentert M, Hagenacker T, Deschauer M, Lingor P, Petri S, Schreiber-Katz O (2021). Informal caregiving in amyotrophic lateral sclerosis (ALS): a high caregiver burden and drastic consequences on caregivers’ lives. Brain Sci.

[20] Cedarbaum JM, Stambler N, Malta E, Fuller C, Hilt D, Thurmond B, Nakanishi A (1999). The ALSFRS-R: a revised ALS functional rating scale that incorporates assessments of respiratory function. BDNF ALS Study Group (Phase III). J Neurol Sci.

[21] Roche JC, Rojas-Garcia R, Scott KM, Scotton W, Ellis CE, Burman R, Wijesekera L, Turner MR, Leigh PN, Shaw CE, Al-Chalabi A (2012). A proposed staging system for amyotrophic lateral sclerosis. Brain.

[22] Balendra R, Jones A, Jivraj N, Knights C, Ellis CM, Burman R, Turner MR, Leigh PN, Shaw CE, Al-Chalabi A (2014). Estimating clinical stage of amyotrophic lateral sclerosis from the ALS Functional rating scale. Amyotrophic Lateral Sclerosis Frontotemporal Degeneration.

[23] Moore A, Young CA, Hughes DA (2019). Health Utilities and Costs for Motor Neurone Disease. Value in Health.

[24] Bundesministerium für Gesundheit [Federal ministry of health] (2018) Online-Ratgeber Pflege. Pflegegrade [Online-guide care. Care levels]. Bundesministerium für Gesundheit. https://www.bundesgesundheitsministerium.de/pflegegrade.html. Accessed Oct 12, 2020

[25] Herdman M, Gudex C, Lloyd A, Janssen M, Kind P, Parkin D, Bonsel G, Badia X (2011). Development and preliminary testing of the new five-level version of EQ-5D (EQ-5D-5L). Qual Life Res.

[26] Van Hout B, Janssen MF, Feng Y-S, Kohlmann T, Busschbach J, Golicki D, Lloyd A, Scalone L, Kind P, Pickard AS (2012). Interim Scoring for the EQ-5D-5L: Mapping the EQ-5D-5L to EQ-5D-3L Value Sets. Value Health.

[27] Von Elm E, Altman DG, Egger M, Pocock SJ, Gøtzsche PC, Vandenbroucke JP (2014). The strengthening the reporting of observational studies in epidemiology (STROBE) statement: guidelines for reporting observational studies. Int J Surg.

[28] Bock JO, Brettschneider C, Seidl H, Bowles D, Holle R, Greiner W, König HH (2015). Calculation of standardised unit costs from a societal perspective for health economic evaluation. Gesundheitswesen.

[29] Institut für Qualität und Wirtschaftlichkeit im Gesundheitswesen [Institute for Quality and Efficiency in Health Care] (2020) Allgemeine Methoden [General methods]. Colone, Germany. https://www.iqwig.de/methoden/allgemeine-methoden-v6-1.pdf. Accessed 13.02.2021

[30] Statistisches Bundesamt [Federal Statistical Office of Germany] (2020) Verbraucherpreisindizies - Gesamtindex und 12 Abteilungen [Consumers' price index - full index and 12 figures]. destatis Statistisches Bundesamt. https://www.destatis.de/DE/Themen/Wirtschaft/Preise/Verbraucherpreisindex/Tabellen/Verbraucherpreise-12Kategorien.html. Accessed 13.01.2021

[31] Icks A, Chernyak N, Bestehorn K, Bruggenjurgen B, Bruns J, Damm O, Dintsios CM, Dreinhofer K, Gandjour A, Gerber A, Greiner W, Hermanek P, Hessel F, Heymann R, Huppertz E, Jacke C, Kachele H, Kilian R, Klingenberger D, Kolominsky-Rabas P, Kramer H, Krauth C, Lungen M, Neumann T, Porzsolt F, Prenzler A, Pueschner F, Riedel R, Ruther A, Salize HJ, Scharnetzky E, Schwerd W, Selbmann HK, Siebert H, Stengel D, Stock S, Voller H, Wasem J, Schrappe M (2010). Methods of health economic evaluation for health services research. Gesundheitswesen.

[32] Statistisches Bundesamt [Federal Statistical Office of Germany] (2021) Einkommen, Einnahmen und Ausgaben in den Gebietsständen [Income, revenues and expenditures in the administration units]. https://www.destatis.de/DE/Themen/Gesellschaft-Umwelt/Einkommen-Konsum-Lebensbedingungen/Einkommen-Einnahmen-Ausgaben/Tabellen/liste-gebietsstaende.html;jsessionid=F71FE5CAC7DB3D3B07816AA772FA4269.internet722. Accessed 01.02.2021

[33] Deutsche Bundesbank [Federal Bank of Germany] (2021) Zeitreihe BBEX3.A.USD.EUR.BB.AC.A04: Euro-Referenzkurs der EZB / 1 EUR = ... USD / Vereinigte Staaten [Time series BBEX3.A.USD.EUR.BB.AC.A04: Euro reference course of the European central bank / 1 EUR =...USD / United States of America]. Deutsche Bundesbank Eurosystem. https://www.bundesbank.de/dynamic/action/de/statistiken/zeitreihen-datenbanken/zeitreihen-datenbank/723452/723452?listId=www_s331_b01012_1&tsTab=0&tsId=BBEX3.A.USD.EUR.BB.AC.A04&id=0. Accessed 28.01.2021

[34] Schöffski O, Schulenburg J-M (2012). Gesundheitsökonomische evaluationen [Health Economics Evaluations].

[35] Zuluaga-Sanchez S, Teynor M, Knight C, Thompson R, Lundqvist T, Ekelund M, Forsmark A, Vickers AD, Lloyd A (2019). Cost effectiveness of nusinersen in the treatment of patients with infantile-onset and later-onset spinal muscular atrophy in Sweden. Pharmacoeconomics.

[36] Statistisches Bundesamt [Federal Office of Statistics] (2020) Sterbetafel 2017/2019—Ergebnisse aus der laufenden Berechnung von Periodensterbetafeln für Deutschland und die Bundesländer [Mortality tables 2017/2019). Wiesbaden. https://www.destatis.de/DE/Themen/Gesellschaft-Umwelt/Bevoelkerung/Sterbefaelle-Lebenserwartung/Publikationen/Downloads-Sterbefaelle/periodensterbetafel-erlaeuterung-5126203197004.pdf?__blob=publicationFile. Accessed 02.10.2022

[37] Acsadi G, Crawford TO, Müller‐Felber W, Shieh PB, Richardson R, Natarajan N, Castro D, Ramirez‐Schrempp D, Gambino G, Sun P, Farwell W (2021) Safety and efficacy of nusinersen in spinal muscular atrophy: The EMBRACE study. Muscle & Nerve:1–10. 10.1002/mus.27187 10.1002/mus.27187 PMC8248061 33501671

[38] Fink JK (2013). Hereditary spastic paraplegia: clinico-pathologic features and emerging molecular mechanisms. Acta Neuropathol.

[39] Miller T, Cudkowicz M, Shaw PJ, Andersen PM, Atassi N, Bucelli RC, Genge A, Glass J, Ladha S, Ludolph AL, Maragakis NJ, McDermott CJ, Pestronk A, Ravits J, Salachas F, Trudell R, Van Damme P, Zinman L, Bennett CF, Lane R, Sandrock A, Runz H, Graham D, Houshyar H, McCampbell A, Nestorov I, Chang I, McNeill M, Fanning L, Fradette S, Ferguson TA (2020). Phase 1–2 trial of antisense oligonucleotide tofersen for SOD1 ALS. N Engl J Med.

[40] Statistisches Bundesamt [Federal Statistical Office of Germany] (2020) Pressemitteilung Nr. 223 vom 19. Juni 2020 [Press announcement no. 223]. https://www.destatis.de/DE/Presse/Pressemitteilungen/2020/06/PD20_223_12411.html. Accessed 31.03.2021

[41] Rosenbohm A, Peter RS, Erhardt S, Lulé D, Rothenbacher D, Ludolph AC, Nagel G (2017). Epidemiology of amyotrophic lateral sclerosis in Southern Germany. J Neurol.

[42] Verhaart IEC, Robertson A, Wilson IJ, Aartsma-Rus A, Cameron S, Jones CC, Cook SF, Lochmüller H (2017) Prevalence, incidence and carrier frequency of 5q–linked spinal muscular atrophy—a literature review. Orphanet Journal of Rare Diseases 12:124: . 10.1186/s13023-017-0671-8 10.1186/s13023-017-0671-8 PMC5496354 28676062

[43] Ruano L, Melo C, Silva MC, Coutinho P (2014). The global epidemiology of hereditary ataxia and spastic paraplegia: a systematic review of prevalence studies. Neuroepidemiology.

[44] Wei Q-Q, Hou Y, Chen Y, Ou R, Cao B, Zhang L, Yang T, Shang H (2021) Health-related quality of life in amyotrophic lateral sclerosis using EQ-5D-5L. Health and Quality of Life Outcomes 19:181: . 10.1186/s12955-021-01822-9 10.1186/s12955-021-01822-9 PMC8290546 34284776

[45] Körner S, Sieniawski M, Kollewe K, Rath KJ, Krampfl K, Zapf A, Dengler R, Petri S (2013). Speech therapy and communication device: impact on quality of life and mood in patients with amyotrophic lateral sclerosis. Amyotroph Lateral Scler Frontotemporal Degener.

[46] Dean R, Jensen I, Cyr P, Miller B, Maru B, Sproule DM, Feltner DE, Wiesner T, Malone DC, Bischof M, Toro W, Dabbous O (2021). An updated cost-utility model for onasemnogene abeparvovec (Zolgensma®) in spinal muscular atrophy type 1 patients and comparison with evaluation by the Institute for Clinical and Effectiveness Review (ICER). Journal of Market Access & Health Policy.

[47] McMillan HJ, Gerber B, Cowling T, Khuu W, Mayer M, Wu JW, Maturi B, Klein-Panneton K, Cabalteja C, Lochmüller H (2021). Burden of Spinal Muscular Atrophy (SMA) on patients and caregivers in Canada. J Neuromuscul Dis.

[48] Belter L, Cruz R, Jarecki J (2020). Quality of life data for individuals affected by spinal muscular atrophy: a baseline dataset from the Cure SMA community update survey. Orphanet J Rare Dis.

[49] Andrews JA, Paganoni S, Braastad C, Cudkowicz M, Atassi N (2014). Disease burden in upper motor neuron syndromes. J Clin Neuromuscul Dis.

[50] De Souza PVS, De Rezende Pinto WBV, De Rezende Batistella GN, Bortholin T, Oliveira ASB (2017). Hereditary spastic paraplegia: clinical and genetic hallmarks. The Cerebellum.

[51] Klimpe S, Schüle R, Kassubek J, Otto S, Kohl Z, Klebe S, Klopstock T, Ratzka S, Karle K, Schöls L (2012) Disease severity affects quality of life of hereditary spastic paraplegia patients. European Journal of Neurology 19:168–171. 10.1111/j.1468-1331.2011.03443.x 10.1111/j.1468-1331.2011.03443.x 21631647

[52] Schreiber-Katz O, Klug C, Thiele S, Schorling E, Zowe J, Reilich P, Nagels KH, Walter MC (2014). Comparative cost of illness analysis and assessment of health care burden of Duchenne and Becker muscular dystrophies in Germany. Orphanet J Rare Dis.

[53] Wetzstein M, Rommel A, Lange C (2015) Informal caregivers - Germany’s largest nursing service. GBE kompakt 6:1–11. 10.17886/RKI-GBE-2016-002

[54] Aranda-Reneo I, Peña-Longobardo LM, Oliva-Moreno J, Litzkendorf S, Durand-Zaleski I, Tizzano EF, López-Bastida J (2020). The burden of spinal muscular atrophy on informal caregivers. Int J Environ Res Public Health.

[55] Thokala P, Stevenson M, Kumar VM, Ren S, Ellis AG, Chapman RH (2020) Cost effectiveness of nusinersen for patients with infantile-onset spinal muscular atrophy in US. Cost Effectiveness and Resource Allocation 18:41: . 10.1186/s12962-020-00234-8 10.1186/s12962-020-00234-8 PMC7539471 33041673

[56] Krauth C, Hessel F, Hansmeier T, Wasem J, Seitz R, Schweikert B (2005). Empirical standard costs for health economic evaluation in Germany—a proposal by the working group methods in health economic evaluation. Gesundheitswesen.

